# Understanding PFAS Behavior: Analysing Contamination Patterns in Surface Water and Sediment of the Apies River, South Africa

**DOI:** 10.1007/s00128-025-04033-w

**Published:** 2025-03-27

**Authors:** R. Okwuosa, P. N. Nomngongo, L. Petrik, O. S. Olatunji, O. J. Okonkwo

**Affiliations:** 1 Department of Environmental, Water and Earth Sciences, Faculty of Science, Tshwane University of Technology, Arcadia Campus, Pretoria, 0001 South Africa; 2 Department of Applied Chemistry, University of Johannesburg, Doornfontein Campus, P.O. Box 17011, Johannesburg, 2028 South Africa; 3 DST/NRF Sarchi Chair: Nanotechnology for Water, University of Johannesburg, Doornfontein, 2028 South Africa; 4 Environmental and Nano Science Group, Department of Chemistry, University of the Western Cape, Cape Town, South Africa; 5 School of Chemistry and Physics, University of Kwazulunatal, Durban, 4000 South Africa

**Keywords:** PFAS, Surface Water, Pollution, Temporal Trends, Sediments

## Abstract

Per- and polyfluoroalkyl substances (PFAS) are persistent environmental contaminants widely detected in water and sediment worldwide. Despite growing concerns about their ecological and health risks, their distribution in African aquatic environments remains understudied. This study addresses the knowledge gap in PFAS contamination by analysing the spatial and temporal distribution of 18 PFAS in Apies River water and sediment in Pretoria, South Africa. Surface water and sediment samples were collected upstream and downstream of the Apies River during dry seasons. The analysis of PFAS concentrations was conducted using liquid chromatography-tandem mass spectrometry. Statistical analysis, including paired t-tests, non-metric multidimensional scaling, and hierarchical cluster analysis, were applied to determine spatial and temporal trends. The study revealed significant spatial variations in PFAS contamination, with upstream locations consistently exhibiting higher concentrations than downstream. In surface water samples, L_PFBS, 4:2 FTS, 6:2 FTS, and L_PFHpS showed statistically significant differences (*p* < 0.05) between sites. Perfluorocarboxylic acids were the dominant PFAS class in surface water (50.47–57.15%), whereas perfluorosulfonic acids were more prevalent in sediments. Upstream sediment had higher L_PFHpS (43.00 ng/g), L_PFDS (38.89 ng/g), and L_PFHxS (23.91 ng/g) than downstream (31.96, 27.84, and 18.02 ng/g, respectively). The findings reveal contamination sources and partitioning between surface water and sediments, aiding in water quality management and pollution mitigation strategies.

## Introduction

Per- and polyfluoroalkyl substances (PFAS), commonly referred to as forever chemicals, are a group of man-made synthetic chemicals that were developed in the mid-1900s (Evich et al., 2022) PFAS encompass a substantial group of fluorinated compounds that are present in certain commercial products and the environment. PFAS exhibit extreme stability, and persistence due to the strength of carbon-fluorine bonds. They exhibit resistance to degradation from natural processes such as exposure to sunlight, microbial activity, and chemical reactions (Zhang et al. 2022). As a result, once released into the environment, PFAS can persist for long periods. Due to their persistence, PFAS can undergo long-range transport through air, soil, and water. Water bodies, including marine waters, estuaries, rivers, streams, and lakes, receive PFAS pollutants through various pathways such as atmospheric deposition, surface runoff, and direct discharge (Sims et al. 2022; Novak et al. 2023). The distribution of PFAS in these water bodies tends to vary based on spatial and temporal locality, as well as the water characteristics and physico-chemical properties of the compounds. PFAS functional group properties influence their fate, transport, and the extent to which they accumulate in the environment (Lyu et al. 2022). In soil and sediment matrices, PFAS are infiltrated through direct application of sludge (Zhou et al. 2024), wastewater irrigation(Shigei et al. 2020) and atmospheric deposition (Gerardu et al. 2023). Anionic PFAS have been reported to have low affinity for soil and sediment particles as stated by Nguyen et al. (2020). This means they do not stick well to soil surfaces and can move more easily through soil, potentially reaching groundwater. In contrast, cationic PFAS are more likely to bind strongly to soils. Beside their functional group, length of the alkyl chain in a PFAS can influence its partitioning behaviour. The studies conducted by Nguyen et al. (2020) showed that the solubility of the PFAS decreased with increasing carbon chain length, while the sorption efficiency increased with increasing carbon chain length.

Despite the global recognition of PFAS contamination and its potential risks to both the environment and public health, there is a significant lack of research focused on the occurrence, distribution, and spatio-temporal trends of these substances in African water systems, particularly in South Africa. Previous studies on PFAS pollution have predominantly centered on regions such as North America and Europe (Viticoski et al. 2022), where comprehensive monitoring systems and regulatory frameworks exist. In contrast, limited research has been conducted on PFAS in South Africa’s water bodies, largely due to inadequate monitoring capacity and a lack of sufficient data to inform effective environmental management strategies. The few studies conducted in Africa have highlighted the presence of PFAS in water systems, such as Lake Victoria in Uganda Arinaitwe et al. (2021). Dalahmeh et al. (2018) analyses wetlands and agricultural areas in Kampala (Uganda), and discovers PFAS in water and other matrices bringing attention to how PFAS have made their way into the food supply and water supplies on land. Legacy PFAS were discovered to be ubiquitous in Nigeria’s surface water, according to research by Adeogun et al. (2024). However, a comprehensive understanding of PFAS dynamics in South African rivers and sediments remains unexplored. This research gap poses a challenge for the development of mitigation and management strategies aimed at reducing PFAS pollution in the region. Therefore, this study aims to address this critical gap by providing a detailed analysis of both legacy and emerging PFAS in surface water and sediment samples from Pretoria, South Africa.

## Materials and Methods

### Chemicals and Materials

18 PFAS standard mix (50 mg/L) listed in the supplementary material (S1) were purchased from Wellington Laboratories (Ontario, Canada). The surrogate mix standards (MPFNA, MPFUdA and MPFHxS) and the isotopically labelled internal mix standards (MPFDA_13C2 and MPFHxA_13C2) were purchased from Wellington Laboratories (Ontario, Canada). LC-MS grade ultrapure water, methanol, acetonitrile, ammonium acetate, and formic acid were purchased from Sigma-Aldrich (South Africa). Supelco ENVI-18™ SPE cartridges (500 mg, 6 ml) were purchased from Sigma-Aldrich (South Africa).

### Sampling

**Fig. 1:**
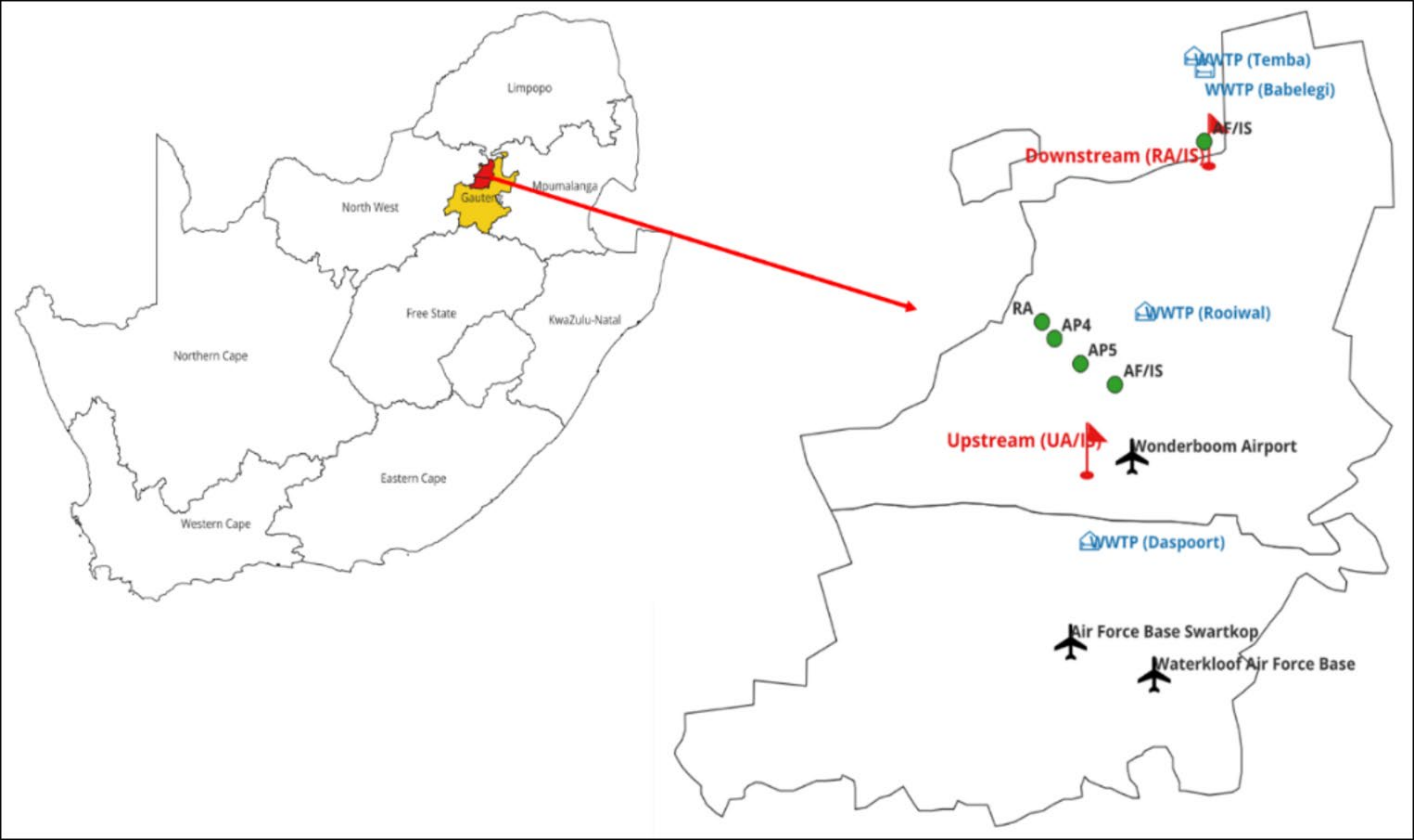
Geographical location of sampling sites within the city of Tshwane

Surface water samples (1 L) were collected in clean high-density polyethylene bottles during the dry season using grab sampling. Samples were collected weekly on days 1, 7, 14, and 21. A field blank (high-density polyethylene bottle with ultrapure water) was used to assess potential contamination from the sampling bottles. Samples were taken from upstream and downstream locations along the Apies River, just below the surface (10–15 cm deep). Three replicate samples were collected from each site. The collected water samples were labelled and stored on ice until transport to the laboratory. Samples were stored in cooler boxes with ice during transit to the laboratory, where they were kept at −4 °C until extraction. Sediment samples were collected upstream and downstream of the Apies River using a Van Veen grab sampler, targeting the top 5–10 cm for recent deposition. Sediment samples were collected from depositional areas near the riverbanks. Three replicates per site were stored in pre-cleaned glass jars with Teflon-lined lids to ensure representativeness and account for spatial variability. Both field blanks and trip blanks were used. Field blanks were prepared with ultra-pure MilliQ water and handled like sediment samples to check for collection contamination. Trip blanks, also containing ultra-pure MilliQ water, were sealed and transported with the samples to assess transport contamination. The collected sediment samples were labeled and stored in ice until transported to the laboratory. The sampling site description is presented in Fig. 1. upstream and downstream locations were defined relative to the flow direction of the Apies River, which flows northward through the city of Pretoria. The upstream site is located south of the city, before significant urban inputs, near military and aviation facilities (Waterkloof Air Force Base and Wonderboom Airport), as well as Daspoort WWTP. The downstream site is positioned further north, beyond major PFAS sources, including Rooiwal, Temba, and Babelegi WWTPs, informal settlements, and industrial areas.

### Sample Pre-treatment and Extraction

#### Sediment and Water Sample Extraction

Sediment samples were air-dried to remove moisture, homogenised using a mortar and pestle, and sieved through a 2 mm sieve to ensure uniform particle size. Approximately 5 g of the homogenised sediment was weighed and placed in a 50 mL polypropylene centrifuge tube. Surface water samples were first filtered using a 0.45 μm glass fiber filter on a vacuum filtration unit to remove suspended matter. Both sediment and water samples were spiked with 250 µL of 100 ng/L labeled surrogate mix standards (MPFNA, MPFUdA, and MPFHxS) and allowed to equilibrate for 1 h. For sediment samples, 20 mL of methanol was added, followed by vortexing for 1 min. The mixture was then sonicated in an ultrasonic bath for 30 min to enhance PFAS extraction and centrifuged at 2000 rpm for 10 min to separate the solid and liquid phases. This extraction step was repeated twice using the same volume of methanol. The combined supernatants were subjected to solid-phase extraction (SPE) cleanup using Supelco ENVI-18™ SPE cartridges (500 mg, 6 mL) to remove impurities and concentrate PFAS. The cartridges were preconditioned with 3 mL methanol followed by 3 mL Milli-Q water, then air-dried before elution of the extracts with 5 mL methanol. For water samples, 250 mL of each sample was passed through Supelco ENVI-18™ SPE cartridges under a vacuum flow rate of 10–15 mL/min. The cartridges were preconditioned with 3 mL methanol followed by 3 mL Milli-Q water, air-dried, and eluted with 5 mL methanol. The eluates from both sediment and water samples were evaporated to dryness under a gentle stream of nitrogen gas. The extracts were reconstituted with 950 µL of methanol and 50 µL of 2000 ng/L isotopically labeled internal mix standards (MPFDA_13C2 and MPFHxA_13C2). After centrifugation, the extracts were transferred to 1 mL vials for liquid chromatography-mass spectrometry (LC-MS) analysis. EPA Method 1633 was employed for all steps of extraction, clean-up, and analysis with minor modifications.

### Instrumental Quantification

PFAS standards were injected and analysed using liquid chromatography-tandem mass spectrometry (Shimadsu LC-MS 8030 triple quadrupole system, Tokyo, Japan). The instrument was equipped with an electrospray ionisation (ESI) source and the target compounds were separated on an inert sustain C18 (3 μm, 2.1 i.d. x 150 mm) HPLC column (Tokyo, Japan). The quantitation of the target compounds was based on the internal standard method calibration with calibration concentrations ranging from 1.0 to 1000 ng/L. A correlation coefficient (R2 ≥ 0.99) was obtained in every internal standard calibration with acceptable precision. The details of the MRM transition for the 18 PFAS compounds with relevant information about their precursor and product ions are presented in the supplementary material (Table S1).

### Quality Assurance and Quality Control (QA/QC)

A series of validation experiments were conducted using various control samples and replicates to confirm the accuracy and consistency of the results. In order to minimise errors and ensure the precision of the data recalibration of instruments and standard operating procedures were implemented. Spiking experiments were performed using known concentrations of analytes to assess the recovery rates and verify the accuracy of the extraction method. The sediment, water and blanks samples were spiked with 250 µL of 100 ng/L of mixed surrogate and the recoveries of each sample calculated. The mean percentage recoveries of the surrogate compounds in the Milli-Q water, sediment and surface water samples, as indicated in Table S2. The mean percentage recoveries ranged from 85.9 to 110.2%, 92.8 to 100.4% and 75.23 to 110.32% for surface water, Milli-Q water and sediment, respectively. The field and trips blanks results were not reported as no contamination was detected during analysis. During instrumental analysis reagent blanks were run between samples to monitor for instrumental contamination for carryover from previous injections. The limit of detection (LOD) and the limit of quantification (LOQ) were calculated using standard deviation of intercepts (S), where LOD and LOQ were defined as 3 and 10 times the S/N ratio, respectively. The values obtained were divided by the dilution factor. The LOD and LOQ values range from 0.012 to 0.016 and 0.012–0.024 ng/L, respectively for water samples. For the sediment samples, the LOD and LOQ values ranges from 0.002 to 0.158 and 0.006–0.476 ng/g, respectively (Table S3).

### Statistical Analysis

All statistical analyses were conducted with using OriginPro 2018 (OriginLab Corporation) software and R using the vegan, pheatmap, ggplot2, and circlize packages. Non-metric multidimensional scaling (NMDS) was applied using Bray-Curtis dissimilarity to visualise differences in PFAS composition across sites and sampling days. Hierarchical cluster analysis (HCA) with Ward’s method was performed to group samples based on PFAS profiles. ANOSIM (Analysis of Similarities) was used to test for significant differences between upstream and downstream sites, while SIMPER (Similarity Percentage Analysis) identified the PFAS compounds contributing most to these differences. Heatmaps were generated to illustrate hierarchical clustering patterns, and boxplots of dissimilarity ranks were created to compare within-group and between-group variations. Stacked bar plots and circular chord diagrams were employed to examine PFAS composition trends and interrelationships over time.

## Results and Discussion

### Spatial and Temporal Distribution of PFAS in Surface Water

**Fig. 2:**
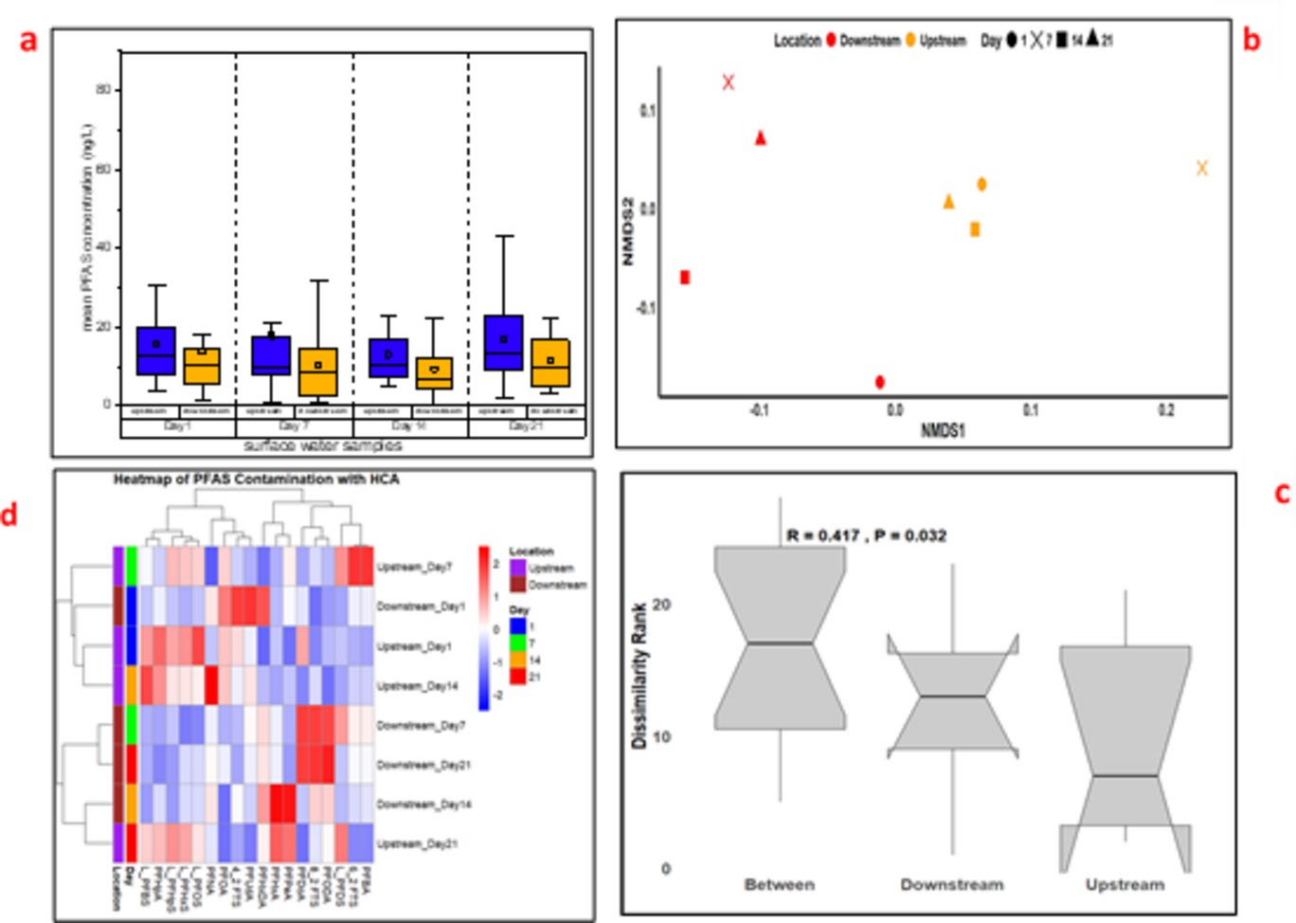
Spatial and temporal distribution of PFAS in surface water. Mean PFAS concentrations (a), NMDS plot of PFAS composition (b), ANOSIM analysis (c), and hierarchical cluster analysis (d)

The PFAS concentration revealed notable upstream and downstream differences across sampling days, with upstream location generally exhibiting higher levels of specific PFAS compounds (Fig. 2a). The mean PFAS concentrations downstream were 13.84, 10.52, 9.41, and 11.79 ng/L, while upstream mean concentrations were 15.80, 17.90, 13.11, and 17.00 ng/L on Days 1, 7, 14, and 21, respectively (Table S4). On Day 1, upstream samples showed significant levels of various PFAS compounds, including L_PFBS (20.63 ng/L), L_PFHpS (18.45 ng/L), L_PFDS (14.33 ng/L), and L_PFHxS (23.26 ng/L) (Table S4). In contrast, downstream concentrations of the aforementioned PFAS were lower, with concentrations of 5.86, 7.40, 3.28, and 11.49 ng/L for L_PFBS, L_PFHpS, L_PFDS, and L_PFHxS, respectively. These concentrations exceeded those reported in Lake Victoria, Uganda, where PFAS concentrations ranged from 0.08 to 23.8 ng/L (Arinaitwe et al. 2021)​.

On day 7, upstream samples showed elevated levels of L_PFDS (36.00 ng/L) and PFBA (69.50 ng/L), while downstream concentrations were 23.80 ng/L for L_PFDS and 14.03 ng/L for PFBA. In this study, PFBA concentrations exceeded levels reported in Lake Tana, Ethiopia, where PFBA was found at an average of 2.9 ng/L (Ahrens et al. 2016)​, but were lower than values recorded in the Vaal River, South Africa, which had PFAS concentrations reaching up to 38.5 ng/L (Groffen et al. 2018). On day 14, upstream samples had increased levels of L_PFBS (23.00 ng/L), L_PFHpS (13.00 ng/L), and PFHpA (41.20 ng/L), while downstream concentrations were significantly lower, with L_PFBS at 2.07 ng/L and L_PFHpS at 4.63 ng/L. On the final sampling day, upstream samples contained high levels of L_PFDS (38.00 ng/L), PFHpA (43.20 ng/L), and PFHxA (24.34 ng/L), whereas downstream concentrations were 9.87, 3.05, and 10.08 ng/L for L_PFDS, PFHpA, and PFHxA, respectively. In this study, PFHpA and PFHxA showed higher concentrations compared to the Nairobi River study, where PFHpA was found at a maximum of 4.49 ng/L and PFHxA at 5.92 ng/L (Chirikona et al. 2022).

To confirm statistical significance, paired t-tests were performed to evaluate differences in upstream and downstream PFAS concentration. The results indicated that L_PFBS, 4:2 FTS, 6:2 FTS, and L_PFHpS exhibited significant spatial variation (*p* < 0.05). This trend is consistent with observations in the Vaal River, where industrial discharges were identified as major contributors to elevated PFAS concentrations (Groffen et al. 2018). However, for other compounds, including PFHxA, PFPeA, PFBA, L_PFOS, PFOA, PFHpA, PFDoA, PFODA, PFUdA, and PFHxDA, no significant differences (*p* > 0.05) were detected between upstream and downstream locations. This observation aligns with findings from Lake Victoria, where PFASs were detected in both urban and rural areas with relatively uniform distribution due to their persistence (Dalahmeh et al., 2018). To further explore spatial contamination patterns, nonmetric multidimensional scaling (NMDS) and ANOSIM analyses were performed (Fig. 2b and c). These multivariate techniques confirmed moderate but significant spatial differences in PFAS composition between upstream and downstream locations (*R* = 0.417, *P* = 0.032). The NMDS plot revealed clustering patterns that align with the paired t-test results, indicating that specific PFAS compounds drive the observed spatial variation. Hierarchical cluster analysis (HCA) revealed distinct upstream-downstream clustering in PFAS contamination (Fig. 2d), supporting the NMDS and t-test findings. The heatmap shows higher contamination in upstream samples, particularly on Day 7 and Day 14, with elevated levels of L_PFBS, PFBA, and L_PFDS. Temporal variation is evident, as some PFAS compounds persist across sampling days, while others fluctuate, suggesting episodic pollution events.

### Spatial and Temporal Distribution of PFAS in Sediment Samples

**Fig. 3:**
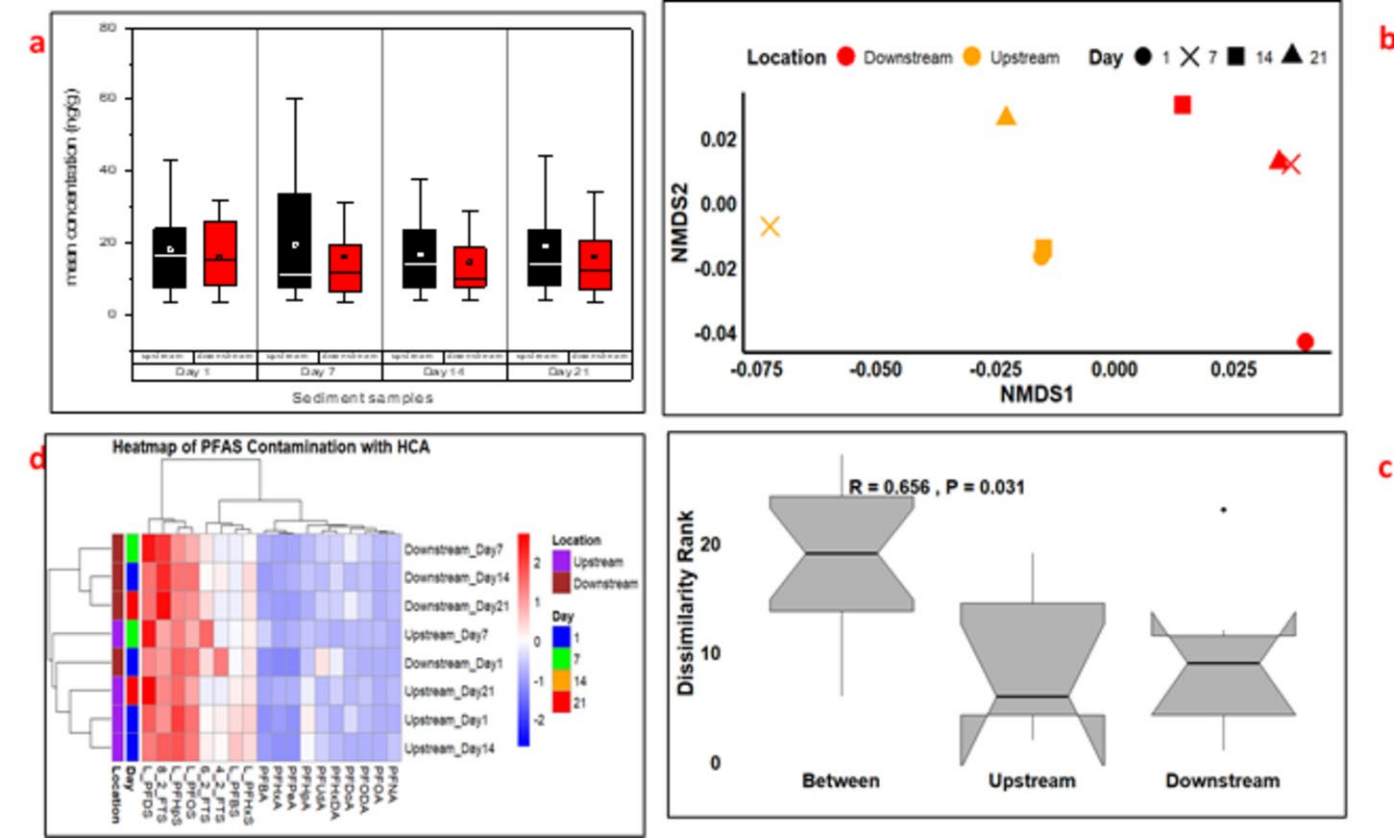
Spatial and temporal distribution of PFAS in sediment samples. Mean PFAS concentrations (a), NMDS plot of PFAS composition (b), ANOSIM analysis (c), and hierarchical cluster analysis (d)

Figure 3 illustrates the spatial and temporal distribution patterns of individual PFAS concentrations in sediment samples collected from upstream and downstream locations on day 1, 7, 14 and 21. The mean PFAS concentrations in sediment samples were consistently higher upstream compared to downstream (Fig. 3a). On Day 1, L_PFBS, L_PFHpS, L_PFDS, and L_PFHxS, with concentrations of 22.59, 43.00, 38.89 and 23.91 ng/g, respectively were higher compared to the other PFAS (Table S2). In contrast, lower concentrations of these compounds were observed downstream: L_PFBS, L_PFHpS, L_PFDS, and L_PFHxS were 15.21, 31.96, 27.84, and 18.02 ng/g, respectively. These concentrations were notably higher than those reported in Lake Hawassa, Ethiopia, where PFOA was the most dominant PFAS in sediment, with mean concentrations ranging from 0.1 to 0.6 ng/g *(*Melake et al. 2022*)*. The elevated upstream concentrations of L_PFDS (60.51 ng/g) and PFBA (11.76 ng/g) on Day 7, compared to downstream levels of 48.36 ng/g and 4.82 ng/g, respectively. These PFBA concentrations exceeded levels reported in Lake Tana, Ethiopia, where PFBA was found at an average of 2.9 ng/g *(*Ahrens et al. 2016*)*but were lower than values recorded in the Vaal River, South Africa, which had PFAS concentrations reaching up to 38.5 ng/g *(*Groffen et al. 2018*)*. Elevated L_PFBS (23.78 ng/g), L_PFHpS (37.56 ng/g), and PFHxA (3.91 ng/g) levels in upstream sediments were reported on Day 14. These patterns are consistent with findings from the Nairobi River Basin, Kenya, where industrial discharge was identified as a major contributor to PFAS accumulation in sediments *(*Chirikona et al. 2022*)*. On the final sampling day, the concentrations upstream were 62.56, 16.94, and 6.11 ng/g for L_PFDS, PFHpA, and PFHxA, respectively, and the concentrations downstream were 34.43, 6.90, and 4.33 ng/g for L_PFDS, PFHpA, and PFHxA, respectively. 8_2 FTS were found in higher concentrations downstream (46.68 ng/g) compared to upstream (38.74 ng/g). These concentrations were comparable to those reported in Nigeria, where PFOS concentrations in sediment ranged from 5.1 to 10.4 ng/g, and PFOA ranged from 0.9 to 4.6 ng/g (Ololade et al. 2018).

To statistically validate these observations, paired t-tests were conducted to assess whether the differences in upstream and downstream PFAS concentrations were significant. The results indicated that L_PFBS, L_PFHpS, L_PFDS, and L_PFHxS exhibited significant spatial variation (*p* < 0.05), suggesting potential sources of contamination upstream, such as industrial discharges or runoff from nearby agricultural areas. This trend is consistent with observations in the Vaal River, where industrial discharges were identified as major contributors to elevated PFAS concentrations (Groffen et al. 2018). However, for other compounds, including PFHxA, PFHpA, and PFBA, no significant differences (*p* > 0.05) were detected between upstream and downstream locations. This observation aligns with findings from Lake Victoria, where PFASs were detected in both urban and rural areas with relatively uniform distribution due to their persistence (Dalahmeh et al., 2018). NMDS and ANOSIM analysis (Fig. 2b and c) further supports this finding, revealing moderate but significant spatial variation (*R* = 0.417, *P* = 0.032), suggesting persistent contamination sources upstream. Hierarchical cluster analysis (HCA, Fig. 2d) grouped samples into distinct clusters based on location and sampling day, reinforcing the upstream-downstream contamination pattern and the impact of local pollution sources on PFAS distribution.

### Percentage Contribution and Distribution Patterns of PFAS Classes in River and Sediment Samples

**Fig. 4:**
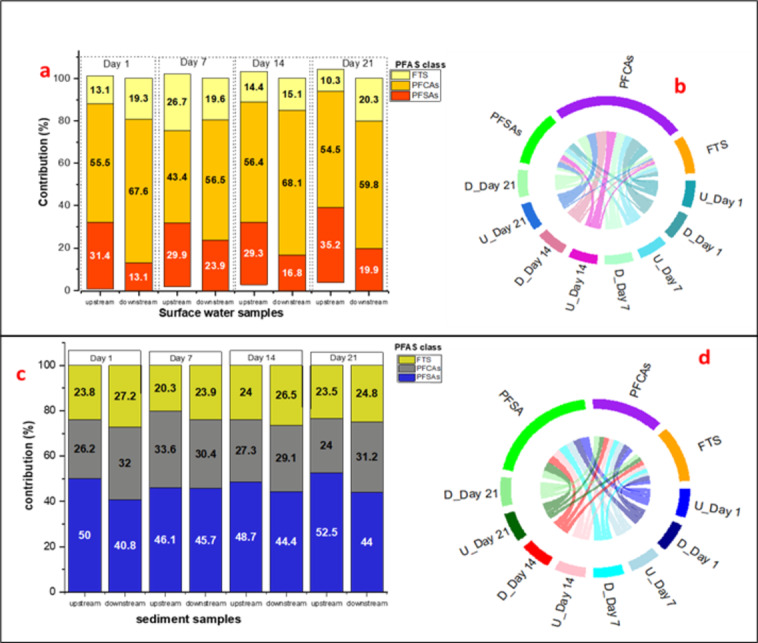
Contribution (%) of each PFAS group (PFCA, PFSA, and FTS) in upstream and downstream surface water samples (left) and sediment samples (right)

The percentage contribution and distribution pattern of PFAS classes in River and sediment samples were analysed using a stacked bar chart and a Chord Diagram, providing complementary insights into the distribution patterns in Upstream and Downstream surface water samples over a 21-day period. The stacked bar chart illustrates the relative contributions (%) of PFSAs, PFCAs, and FTS in each sample location across different days, highlighting clear differences between Upstream and Downstream sites. The percentage contribution of PFAS classes (PFSAs, PFCAs, and FTS) in surface water samples exhibited distinct spatial variations between Upstream and Downstream locations (Fig. 4a). PFCAs were the dominant PFAS class across all sampling days, showing a significant increase in Downstream samples (56.48–68.08%) compared to Upstream (43.37–56.35%). PFSAs were more prevalent in Upstream samples (29.28–35.23%) but significantly lower Downstream (13.14–23.90%). Similarly, FTS contributions ranged from 10.32 to 26.73% in Upstream and 15.11–20.33% in Downstream samples. This trend aligns with Ahrens et al. (2016), who found that PFCAs were predominant in surface water (68%) in Lake Tana, Ethiopia. Similar findings were reported by (Zheng et al. 2017) in the Yangtze River, where PFCAs were found to dominate PFAS profiles due to their resistance to adsorption and degradation.

The chord diagram (Fig. 4b) further illustrates how PFAS groups were spatially distributed between upstream and downstream surface water samples. In the chord diagram, the left half displays sampling locations: U_Day 1, U_Day 7, U_Day 14, U_Day 21 (upstream samples) and D_Day 1, D_Day 7, D_Day 14, D_Day 21 (downstream samples). The right half represents PFAS chemical groups: PFSAs (green), PFCAs (purple), and FTS (orange). The colored ribbons (connection) indicate the relative contributions of each sampling location to the different PFAS groups; thicker ribbons signify higher PFAS concentrations at the respective sites. Predominant connections of a PFAS group to either upstream or downstream locations suggest higher concentrations in those areas, offering insights into PFAS distribution patterns in surface water. The stronger linkages between PFCAs and downstream samples suggest that these compounds remain in the water column and are transported downstream with minimal retention in sediments. This pattern is attributed to the low adsorption tendency of PFCAs compared to PFSAs and FTS, as PFCAs have weaker interactions with sediment particles (Gao et al. 2020). In contrast, PFSAs and FTS exhibit stronger adsorption properties, leading to higher concentrations in Upstream regions where sediment deposition is more dominant. This finding aligns with previous studies that reported higher PFCAs in surface water due to their increased mobility, whereas PFSAs and FTS tend to associate with sediments (Meng et al. 2021).

The percentage contribution of the PFAS groups including in sediment samples varied over different sampling days. The percentage of PFSAs ranged from 46.10 to 52.53% in upstream, while in downstream PFSAs ranged from 40.84 to 45.66%. The percentage of PFCAs in upstream samples was between 20.27 and 24.01%, compared to downstream percentages that ranged from 23.95 to 27.17%. FTS contributions in upstream samples varied from 23.99 to 33.624%, whereas downstream, they ranged from 29.131 to 31.981%. Over the sampling days, sediment samples from both upstream and downstream locations exhibited a higher percentage of PFSAs compared to PFCAs and FTS. In contrast, PFCAs have weaker adsorption tendencies, which results in their higher prevalence in surface water. The dominance of PFSAs in sediments is consistent with Gao et al. (2020), who found that PFSAs had a strong affinity for sediment. The Chord Diagram shown in Fig. 4d illustrates the relationship between sampling locations (sediment Upstream/Downstream at different time points) and PFAS classes. The dominance of PFSAs in sediment samples is evident from their strong connections, particularly in later sampling days (Day 14 and 21), suggesting accumulation over time. Conversely, PFCAs show broader distribution, indicating their greater mobility in sediments, while FTS connections appear weaker, suggesting lower retention.

### Classification According to Chain Length in Sediment and Surface Water Samples

**Fig. 5:**
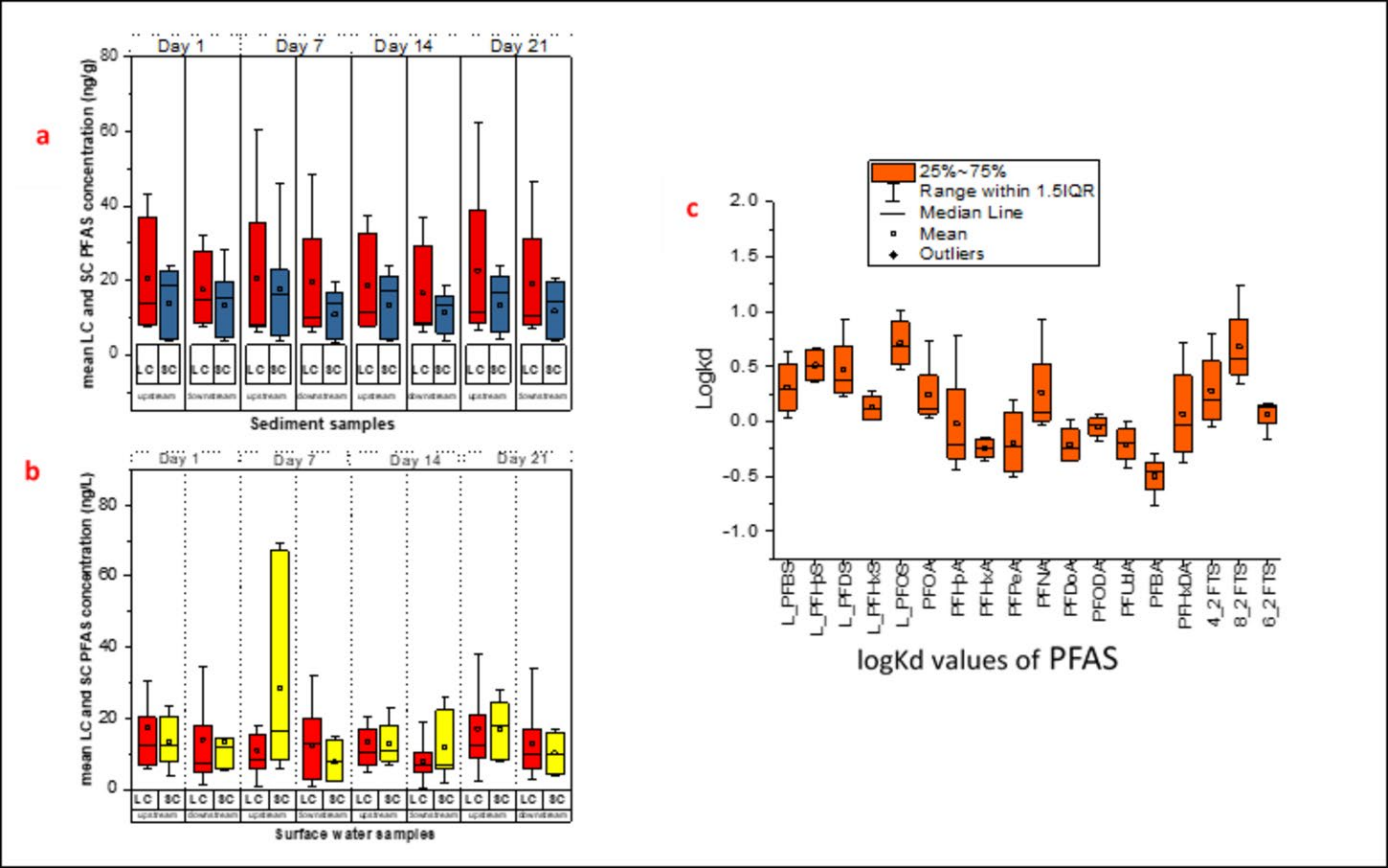
The classification of PFAS by chain length in sediments (a), surface water (b) and logKd values of PFAS (c)

Figure 5 compares the mean concentrations of short-chain and long-chain PFAS in sediment samples (a) and surface water samples (b) on Days 1, 7, 14, and 21. The mean concentrations of short-chain PFAS in upstream sediment samples were 13.96, 17.76, 13.38, and 13.58 ng/g, and in downstream sediment samples were 13.44, 10.92, 11.32, and 11.80 ng/g on Days 1, 7, 14, and 21, respectively (Table S6). The mean concentrations of long-chain PFAS in upstream sediment samples were 20.75, 20.60, 18.79, and 22.61 ng/g, and in downstream sediment samples were 17.50, 19.55, 16.57, and 19.06 ng/g on Days 1, 7, 14, and 21, respectively. Paired t-tests results indicate that there is no statistically significant difference (*p* > 0.05) between the concentrations of short-chain PFAS in upstream and downstream sediment samples over the 21-day period.

Long-chain PFAS concentrations were significantly different (*p* < 0.05) between the upstream and downstream locations upstream over the 21-day period. The concentrations of long-chain PFAS were generally higher than the short-chain PFAS in sediment samples. Similar findings were reported by Nguyen et al. (2020). The authors reported that short-chain PFAS are more susceptible to desorption and leaching from soils than long-chain PFAS. The mean concentrations of short-chain PFAS in upstream water were 12.42, 31.38, 11.15, and 16.76 ng/L, and in downstream water were 14.75, 8.37, 13.43, and 11.01 ng/L on Days 1, 7, 14, and 21, respectively (Table S6). The concentrations of long-chain PFAS in upstream water were 17.07, 10.36, 13.30, and 16.77 ng/L, and in downstream water were 14.74, 12.92, 8.24, and 13.43 ng/L on Days 1, 7, 14, and 21, respectively. The results of the paired t-tests for short-chain and long-chain PFAS concentrations indicates that there is no statistically significant difference (*p* > 0.05) between the concentrations of short-chain PFAS in upstream and downstream water samples over the sampling period. The concentrations of short-chain PFAS are relatively stable between the two locations. On the other hand, long-chain PFAS concentrations are significantly different (*p* < 0.05) between the upstream and downstream locations.

​ The behavior of PFAS partitioning between the water and sediment phases is illustrated in Fig. 4c. The observed distribution coefficients (logKd) values demonstrate the tendency of short-chain and long-chain PFAS to adsorb to sediments versus remaining in the aqueous phase. Short-chain PFAS like L_PFBS, PFHxS, and PFOA have lower logKd values, suggesting they have a greater affinity for the water phase and are more mobile in aquatic environments. For example, L_PFBS had a logKd of 0.039 on Day 1, which increased to 0.635 by Day 21. In contrast, PFHxS maintained a consistently low logKd value, ranging from 0.012 to 0.271 throughout the study period (Table S7). These observations are consistent with previous research indicating that short-chain PFAS have lower soil–water partitioning coefficients, resulting in higher mobility and persistence in water bodies (reference). This trend aligns with Chirikona et al. (2022) study, where logKd ranged from 2.5 (PFUdA) to 4.9 (PFOS), confirming greater sediment affinity. However, logKd values in this study (mean = 0.13) were much lower than those reported by Chirikona et al.(2022). Dissimilarities in sediment composition might explain this disparity. The lower logKd values suggest a higher risk of groundwater contamination, as PFAS remain mobile in the aqueous phase, whereas the higher logKd values show that sediments act as a primary reservoir for PFAS, resulting in long-term environmental persistence.

### Identifying PFAS Sources in Water and Sediment Samples

**Fig. 6:**
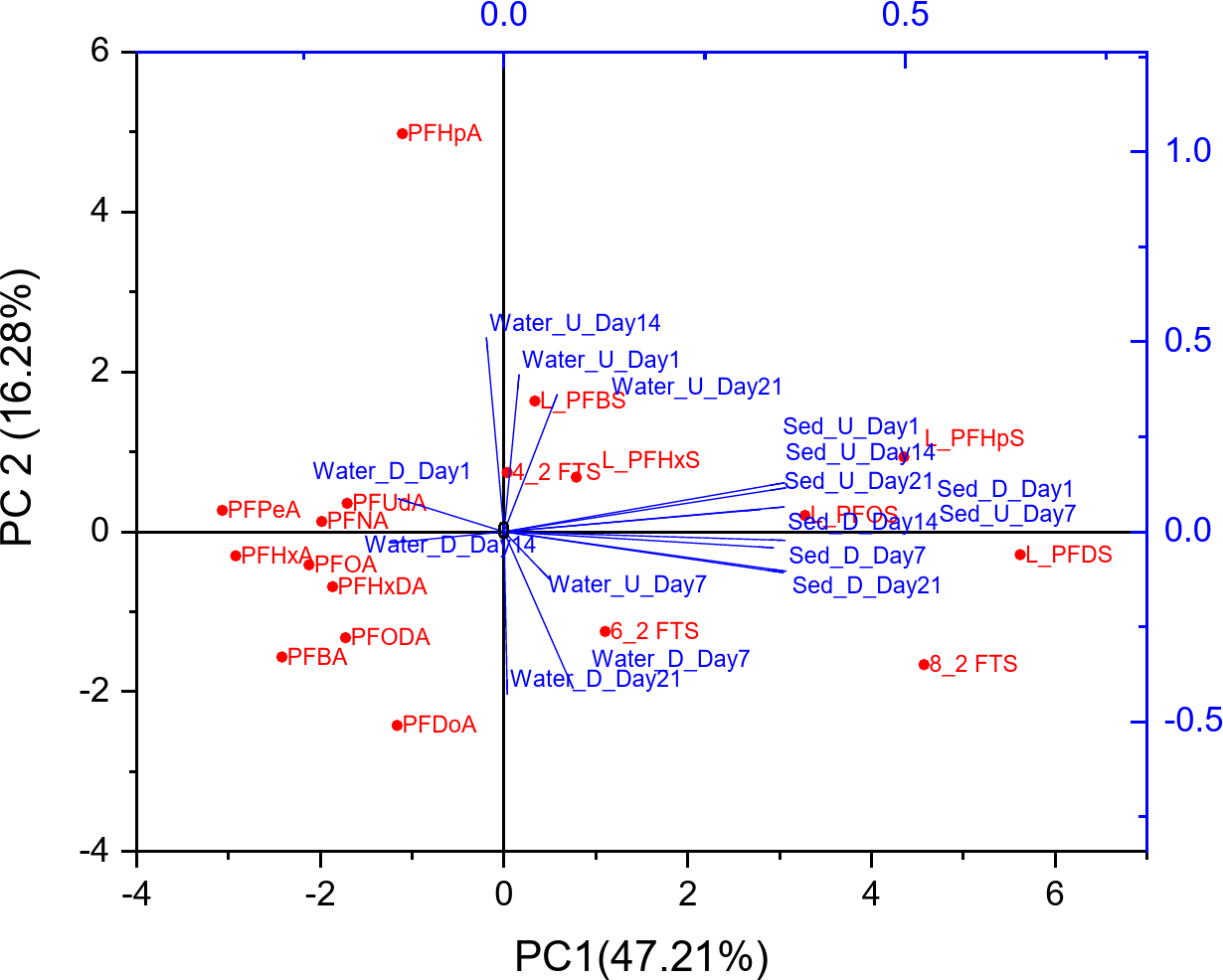
PCA Biplot of PFAS Contamination in Water and Sediment Samples

Principal Component Analysis (PCA) was used to assess the distribution and potential sources of PFAS contamination in both water and sediment samples from the Apies River. The PCA biplot (Fig. 6) revealed a clear distinction between upstream and downstream contamination patterns, suggesting multiple pollution sources.

On day 1, the PCA clustering shows that L_PFBS, L_PFHpS, L_PFDS, and L_PFHxS are strongly associated with upstream sites. Both water and sediment samples from upstream exhibited higher concentrations of mentioned PFAS compared to downstream. This pattern indicates localised sources upstream, likely linked to aqueous film-forming foam (AFFF) used at Waterkloof Air Force Base and Wonderboom Airport, as well as industrial activities. The co-occurrence of L_PFDS and fluorotelomer sulfonates (FTS) in upstream samples suggests a shared contamination source, likely linked to AFFF from military and aviation facilities. On Day 7, L_PFDS and PFBA remained prominent in upstream locations. L_PFDS, being a long-chain PFAS, remained associated with sediment samples, while PFBA showed stronger correlations with water samples, reflecting its higher mobility and potential urban wastewater origins. By Day 14, the PCA shows that PFHpA and PFHxA cluster with sediment samples, reinforcing their industrial origins. These findings align with previous studies identifying industrial discharges from textile, metal plating, and chemical manufacturing activities as contributors to PFAS accumulation in sediments(Chirikona et al. 2022). On Day 21, 8_2 FTS exhibited an inverse trend, with higher concentrations downstream than upstream. The PCA confirms its stronger presence in downstream sediments, suggesting a secondary input from WWTP effluent or sediment resuspension. The PCA results confirm that upstream PFAS contamination is primarily driven by AFFF use and industrial discharges, while downstream contamination is influenced by WWTP effluents and urban runoff. This trend is consistent with observations in the Vaal River, where industrial discharges were identified as major contributors to elevated PFAS concentrations (Groffen et al. 2018). These findings highlight the need for enhanced monitoring of military and industrial sites upstream, as well as improved wastewater treatment processes to reduce PFAS transport and accumulation downstream.

## Conclusion

The PFAS concentrations observed in this study exceeded several established regulatory guidelines for both water and sediment. While international thresholds for PFAS in sediment remain limited, the European Union’s proposed environmental quality standard (EQS) for PFOS in sediment is 13.5 ng/g, a value that was significantly surpassed in this study, particularly for L_PFDS, L_PFHpS, and 8_2 FTS. Similarly, for water, PFAS concentrations in surface water reported in this study exceeded the European Commission’s recommended limit of 4.4 ng/L. If water concentrations in this study approached these levels, they would indicate substantial environmental and potential human health risks. These high levels highlight the need for stricter monitoring and intervention strategies to mitigate PFAS contamination, particularly in upstream locations where persistent sources are evident. To effectively manage PFAS pollution, upstream sources such as WWTP effluents, agricultural runoff, and informal settlements should be prioritized in regulatory frameworks. Improved wastewater treatment technologies and agricultural best management practices could significantly reduce PFAS input into the river system. Short-chain PFAS exhibited high mobility, with no significant differences (*p* > 0.05) between Upstream and Downstream concentrations. Long-chain PFAS showed significant differences (*p* < 0.05), decreasing Downstream, indicating stronger adsorption onto particulates and sediments. Log Kd values confirmed that short-chain PFAS remained in water, while long-chain PFAS preferentially partitioned into sediments. PFCAs were the dominant PFAS class in river water with significantly higher concentrations Downstream compared to Upstream. PFSAs were the dominant PFAS group in sediment samples. The study limitations include the relatively short sampling period and the potential variability in PFAS concentrations due to seasonal changes and other environmental factors. Additionally, the sampling frequency and spatial resolution could be improved in future studies to provide a more comprehensive understanding of PFAS dynamics.

## Data Availability

All the data generated or analysed during this study are included in this article.
